# Racial disparities in cancer care, an eyeopener for developing better global cancer management strategies

**DOI:** 10.1002/cnr2.1807

**Published:** 2023-03-27

**Authors:** Bharmjeet, Asmita Das

**Affiliations:** ^1^ Department of Biotechnology Delhi Technological University Delhi 110042 India

**Keywords:** cancer care, cancer health disparities, cancer management, cancer therapy, racial disparities

## Abstract

**Background:**

In the last few decades, advancements in cancer research, both in the field of cancer diagnostics as well as treatment of the disease have been extensive and multidimensional. Increased availability of health care resources and growing awareness has resulted in the reduction of consumption of carcinogens such as tobacco; adopting various prophylactic measures; cancer testing on regular basis and improved targeted therapies have greatly reduced cancer mortality among populations, globally. However, this notable reduction in cancer mortality is discriminate and reflective of disparities between various ethnic populations and economic classes. Several factors contribute to this systemic inequity, at the level of diagnosis, cancer prognosis, therapeutics, and even point‐of‐care facilities.

**Recent Findings:**

In this review, we have highlighted cancer health disparities among different populations around the globe. It encompasses social determinants such as status in society, poverty, education, diagnostic approaches including biomarkers and molecular testing, treatment as well as palliative care. Cancer treatment is an active area of constant progress and newer targeted treatments like immunotherapy, personalized treatment, and combinatorial therapies are emerging but these also show biases in their implementation in various sections of society. The involvement of populations in clinical trials and trial management is also a hotbed for racial discrimination. The immense progress in cancer management and its worldwide application needs a careful evaluation by identifying the biases in racial discrimination in healthcare facilities.

**Conclusion:**

Our review gives a comprehensive evaluation of this global racial discrimination in cancer care and would be helpful in designing better strategies for cancer management and decreasing mortality.

## 1 INTRODUCTION

Despite coherent efforts that have led to a significant reduction in cancer mortality, it remains to be the second major cause of death, following cardiovascular diseases. With 215 deaths from cancer per 100000 individuals, the mortality rate peaked in 1991.^1^ At the beginning of the present year, the American Cancer Society estimated a total of 1918030 collective cancer cases with 609360 deaths in 2022^2^ and in 2020 there were about 10 million cancer deaths and an anticipated 19.3 million additional cancer incidences worldwide.^3^ Cancer prevalence and cancer mortality are fortunately dropping in the world due to efficient healthcare facilities, better monitoring, early detection, and better cancer management. However, some populations continue to exhibit a greater risk of predominance and mortality concerning specific types of malignancies. Human populations all over the world are impacted by cancer but certain types of cancer are predominant in certain geographical locations.^4^ Various factors have been attributed to the impact of this skewness, that include genetic,^5^ socioeconomic,^6^, ^7^ and environmental factors.^8^ The National Cancer Institute (NCI) specifies cancer health disparities as discrepancies in disease metrics, namely the occurrence rates, death rates, complications, survival rates, budgetary stress, and living standards [Courtesy: Cancer Disparities—NCI]. There appears to be a huge disparity in screening and early detection of cancer and the choice of treatment that is predominant among population subgroups. Disparities are apparent in the fact that although overall results show increasing awareness, better screening facilities, and significantly improved cancer mitigation, certain subgroups are not seeing the same gains as other groups. Such observations require a better understanding of the factors responsible for such differential mortality rates and improved cancer management and call for designing strategies for better implementation of the same.

These disparities are the outcome of complex and interconnected factors, making it challenging to separate them and analyze each factor's independent relative impact. Major cancer health disparities and associated mortality that have been noticed in different sections of the population are related to geographical locations, socioeconomic status, and genetics.^5^, ^9^ There is a large regional variation in both cancer cases, kind, and disease prognosis.

**FIGURE 1:**
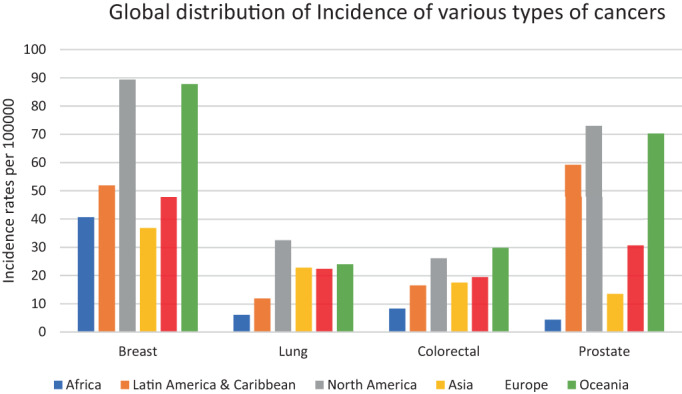
Incidence rates of common types of cancers in different ethnicities per 100 000. Blue, orange, gray, yellow, red, and green bars represent incidence rates per 100 000 for African, Latin American & Caribbean, North American, Asian, European, and Oceanic populations respectively for Breast, Lung, Colorectal, and Prostate cancers. *Source*: Data adapted from GLOBOCAN, 2020.^10^

Breast cancer accounts for 25% of all women screened and leads to 16.6% of deaths due to cancer.^3^ Incidence of breast cancer is significantly higher in developed countries like North America and Oceania (Figure 1). The proportion of risk factors for breast cancer has been known to be impacted by significant alterations in diet, lifestyle, sociocultural, and architectural environments brought on by developing countries and an increase in the number of women in the industries. Lung cancer represents 11.4% of total cancer cases.^3^ The incidence of lung cancer as seen in Figure 1 (the primary data to synthesize the following secondary data was obtained from GLOBOCAN, 2020) is reflective of greater exposure to pollutants and is an unfortunate outcome of industrialization, hence Africa and Latin America exhibit relatively lower incidences. The prevalence of colorectal cancer is about 10% of the total incidences of cancers.^3^ Incidence rates of colorectal cancer in North America and Oceania are higher than in others due to the predominance of junk food in the diet and a sedentary lifestyle. Heavy alcohol use, tobacco consumption, and intake of red or processed meat are other contributing risk factors. Prostate cancer, being one of the most diagnosed cancers in men accounts for variable frequencies in incidence worldwide. Latin America, North America, Europe, and Oceania show greater incidence predominantly due to regular monitoring and marker‐based screening. The greatest incidence rates for prostate cancer among black males are found in the Caribbean and the United States.^11^

**FIGURE 2:**
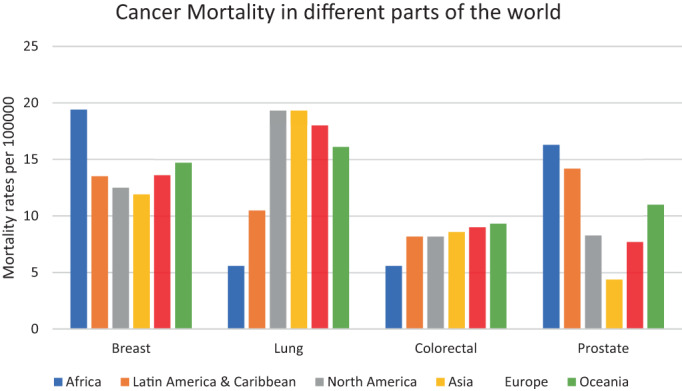
Mortality rates of common types of cancers in different ethnicities per 100 000. Blue, orange, gray, yellow, red, and green bars represent incidence rates per 100 000 for African, Latin American & Caribbean, North American, Asian, European, and Oceanic populations respectively for Breast, Lung, Colorectal, and Prostate cancers. *Source*: Data adapted from GLOBOCAN, 2020.^10^

A higher mortality rate due to breast cancer in the African population, despite low incidence (Figure 2) exemplifies the fact that due to a lack of regular screening early detection of breast cancer does not take place, resulting in higher mortality rates. Lung cancer is a very aggressive cancer and hence leads to higher mortality in all populations with a higher incidence of the disease. The incidence and consequently, mortality rates are highly impacted by the state of industrialization and exposure to associated pollutants. Mortality rates due to colorectal cancers are more or less similar in all regions; the underlying reason for this could be urbanization, dependence on processed foods, a sedentary lifestyle, and lack of physical activities. Prostate cancer is curable if detected early, and hence, the lack of early detection of cancer in the African population due to a lack of effective screening facilities results in higher mortality rates as compared to North America, where the mortality rate is low despite a higher incidence, as seen in the graph.

Factors like insufficient knowledge, poverty, and health insurance appear to be equally important when compared to biological factors in accessing early diagnosis and appropriate treatment.^12^ Additionally, societal injustices like the lingering effects of racial discrimination still have an impact on how patients and doctors connect.^13^ Furthermore, cultural traits may influence how people behave in terms of their health management including regular health monitoring, prophylactic treatment, and whether they trust conventional medicine over alternative types of treatment.^14^

Reducing cancer deaths and increasing survival among the underprivileged constitute the eradication of inequities.^12^ Our understanding of the mechanism and parameters contributing to the reduction in this disparity are inadequate due to the dearth of data, with regards to cancer mitigation and medical & palliative support. To overcome this shortcoming, large cohort studies are needed to be conducted and meta‐analysis of the disparities in a fatality in various ethnic populations needs to be investigated more comprehensively. However, such widespread analyses are very limited due to prohibitive costs and lack of sensitivity. Our review aims to portray a comprehensive analysis of multiple parameters that are crucial in influencing disparities in cancer mortalities in different racial populations with an intent to suggest better mitigation strategies for cancer management across different sections of society.

## 2 DISPARITIES DUE TO SOCIAL DETERMINANTS

Incidence, mortality, and risk factors for cancer vary not just by race and ethnicity but also by socioeconomic levels.^15^, ^16^ Indigence, culture, and societal injustice are socioeconomic factors that influence the discrepancy in deaths due to cancer.^17^ A significant societal factor causing health disparities is poverty.^18^ Tobacco use, inadequate diet, idleness, and being overweight are additional cancer hazard factors connected to socioeconomic disparities. Tobacco corporations frequently use poor and minority groups as sales targets. These groups frequently lack access to appropriate nutrition and fresh foods, as well as few possibilities for appropriate recreational body exercises.^19^

Research indicates that racial differences in the incidence of breast cancer seems less pronounced while there is a significant difference in the mortality rates in different ethnic groups. Social and economical aspects impact the choice of treatment and cancer management so significantly that the cancer outcome shows the immense disparity in ethnic populations.^20^ Irrespective of ethnicity, poverty is linked to worse breast carcinoma results for all; nevertheless, because more black Americans compared to white populations are poor and are, therefore, very prone to exhibiting higher death rates.^21^ Low income and unavailability of insurance coverage deter women from regular breast cancer screenings, resulting in higher chances of detection at a later stage resulting in a greater risk of mortality. Similarly, African and Asian women hardly visit a healthcare practitioner regularly, mammography frequencies are lower and the likelihood of early‐stage detection is low.^22^ Additionally, prohibitive costs often get subpar and unsuitable treatment increases the risk of death in these patients.^15^ Recent advances in monoclonal antibody‐based therapies specifically administered to breast cancer patients based on their marker profile have resulted in an immense increment in cancer regression in patients unresponsive to conventional treatments.^23^ However, such treatments are expensive and often require advanced medical facilities which are unavailable to a great majority of women, the world over. A majority of the world population lack or have insufficient health insurance and relies on governmental interventions. Globally, women in various countries are living in locations with poor infrastructure, which makes it difficult for them to reach basic service facilities and doctors for diagnosis, treatment, or even follow‐ups.^24^ Genetic profiling for the determination of propensity to certain forms of cancer and prophylactic vaccination for cancer is followed in less than 1% of the global population and is also disparate in different ethnic groups. This is often due to a lack of awareness and also due to social deterrents.

Cardiovascular disease, diabetes, hypertension, obesity, and respiratory illness are more common comorbidities among low‐income women, which restricts their treatment choices.^25^ Black females are more inclined than white females to consume a diet rich in fat, deficient, in vegetables and fruits, and are less likely to engage in routine exercise, therefore, more prone to be overweight.^26^ The discrepancy in breast carcinoma rates among females is, therefore, affected by nutritional and lifestyle factors that are indirectly linked to socioeconomic constraints. Further ignorance of disease, lower levels of education, and religious and cultural taboos are additional factors that often lead to late‐stage diagnosis and inappropriate treatments, leading to deaths.^27^ Contrary to white women, black women are more likely to depend on supernatural and spiritual intervention, instead of getting the proper medical care, which can be harmful to their survival.^28^ In general, societal injustice, poverty, and other variables play a direct and indirect role in the gap in breast carcinoma rates among females. Similar socioeconomic discrepancies are also observed in various developing nations including India.^29^ Interestingly in India, there is a steep rise in incidences of breast cancer in urban women, mainly due to stress, lifestyle choices, late pregnancies, and late menopause. Among rural women, breast cancer incidences are however, lesser as compared to their urban counterparts, although there is a higher incidence of cervical cancer among them.^30^, ^31^ In rural India, the constraints leading to cancer mortality are often a lack of early diagnostics, advanced facilities and health care options. Hence, it is obvious that prudent cancer management is not only achievable by addressing economical and infrastructural constraints but also requires a holistic understanding of the factors in specific ethnic populations in addition to the uplifting of care facilities.

The unavailability of exposure to high‐quality healthcare and therapeutic trials is considered to be the main cause of racial discrepancies in lung carcinoma survival.^32^ It is significant to note that social determinants of wellness may contribute to differences in lung carcinoma therapy. These include, (1) both social and economic considerations, such as having health insurance or having the capacity to spend money for treatment, which affects the uninsured and disadvantaged populations, which comprises of many impoverished populations, in terms of access to effective adjuvant therapies.^33^, ^34^ (2) Lack of healthcare awareness, and literacy levels have an impact on the patient's choice of treatment and decisions of adherence and follow‐ups for the treatment which are often prolonged. Poor healthcare awareness will probably have an impact on how well lung carcinoma sufferers comprehend their condition and can handle their respective treatment plans. (3) Improper patient treatment decisions are often the consequence of mistrust of the medical profession, which is a result of their past interactions with the medical system. Negative surgical views, fatalism, and skepticism have been put out as possible explanations for why certain patients are less able to adhere to and get prescribed therapy.^35^ Mistrust is often fuelled by a dearth of knowledge about advancements in ethical principles controlling healthcare and the substandard treatment delivered in unregulated healthcare facilities. (4) Therapeutic inequities may be related to localities or neighborhoods having insufficient practical availability or usage of therapeutic facilities. People living in remote versus metropolitan locations, or living in a community with a high or low socioeconomic status are all connected to the lack of sufficient diagnostic and therapeutic facilities.

Colorectal cancer (CRC) is the third most typical cancer in the United States, irrespective of gender.^36^ The chances of men getting CRC are more than women (4.3% vs. 4%). Age and hereditary risk factors are just two of the several variables that have been found to influence CRC development risk.^37^, ^38^, ^39^ Being an aging‐related condition, the probability of CRC increases with a person's age; according to recommendations, those with medium probability should begin screening tests at 50 years of age.^37^ However, there are complicated correlations between race/ethnicity, socioeconomic status (SES), and CRC.^39^, ^40^ Poor diet and a sedentary lifestyle are two modifiable factors linked to CRC risk that is also linked to SES. Lifestyle choices may have an impact on the microbiota and biological behavior of colonic stem cells as well as the regional colonic environment.^41^ A balanced diet, hormone replacement therapy, and aspirin prescription or NSAIDs may all lower the risk of CRC; exposure to these elements may also be correlated with SES and accessibility to healthcare. SES‐related elements like income, education level, and medical insurance have an impact on who has access to resources and services for healthcare.^39^

It is well‐accepted that social and economic variables influence prostate cancer occurrence. Prostate cancer risk frequently has an opposing relationship with social and economic position. Poor SES is linked to a reduced likelihood of surviving or quality of life. Based on socioeconomic background, race, level of education, and unemployment, prostate cancer survival vary dramatically. The adverse relationship between social assistance and the advanced stage of prostate cancer detection may be explained by several causes.^42^, ^43^, ^44^, ^45^ Men may be persuaded to get screened for prostate malignancy by their partner, other family members, or friends in their network. Married men are better at prostate cancer management due to early screening and better therapy than unmarried men.^46^

## 3 DISPARITIES IN DIAGNOSIS

**FIGURE 3:**
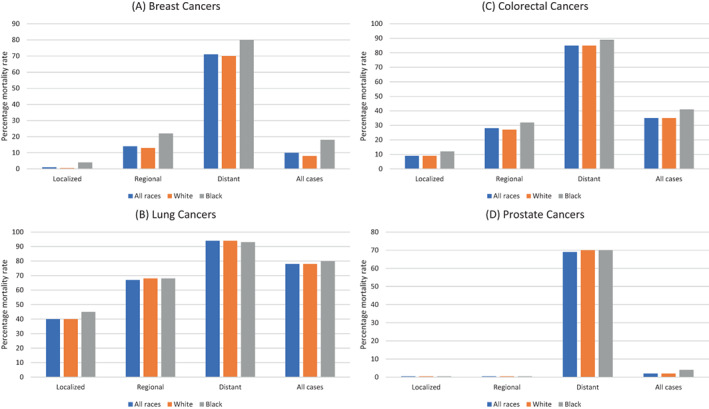
Percentage mortality rate for US patients with respect to different stages of cancers. (A) Breast cancers, (B) Lung cancers, (C) Colorectal cancers, and (D) Prostate cancers. Cancer is classified as localized, regional, or distant depending on the site of the disease. Localized refers to a disease that is limited to the site of origin, regional refers to cancer that has spread to an adjacent area, and distant refers to the post‐malignancy stage of cancer. Mortality rates are shown in blue for all races, orange for white patients, and gray for black patients. *Source*: Graphs adapted from a study by Siegel et al.^2^

While Asian women often get breast cancer between the ages of 40 and 50, Non‐Hispanic White women typically develop it between the ages of 60 and 70.^47^ It is estimated that genetic factors account for 5%–10% of breast carcinoma cases.^48^ The majority of autosomal dominant hereditary breast malignancies are resulting from alterations in BRCA genes (1 and 2), which are present at the 17th and 13th chromosomes, respectively. Human genes called BRCA1 and BRCA2 translate into tumor suppressors, which contribute to DNA repair as well as aid in preserving the integrity of the genomic information. Whenever they are modified, it results in DNA damage and mutation and cells are more prone to further genetic changes that might result in the establishment of cancer. Depending on one's race and ethnicity, these alterations might occur more, or less frequently. For instance, Ashkenazi Jewish females (8.3%) had the greatest incidence of BRCA1 mutations. Hispanic females (3.5%), non‐Hispanic white females (2.2%), Black females (1.3%), and Asian females (0.5%) are next.^49^ Asian women are unlikely to undergo regular breast cancer screening as advised by WHO^49^ and this may be the cause of the historically lower incidence of Breast cancer among Asian women, compared to their western counterparts. 55%–65% of BRCA1 mutation‐containing females and 45% of BRCA2 mutation‐containing females have a probability of developing breast cancer after the age of 70 years. Additionally, before 70 years of age, ovarian cancer may manifest in 39% of females with detrimental BRCA1 alteration and 11%–17% of females with BRCA2 alteration.^50^ Despite the fact that deleterious BRCA 1 and BRCA 2 mutations are known to result in breast carcinoma in greater than 50% of the households with recurrent cases, mutations in other genes have also been associated with increased risks of the disease.^51^, ^52^ ATM, BRIP1, CHEK2, CDH1, MLH1, MLH2, MRE11A, NBN, PTEN, PALB2, RAD50, RAD51C, SEC23B, STK11, and TP53 genes all have rare mutations. By the time they are 70 years old, 33% of females who have a dangerous PALB2 gene alteration will have breast cancer. Those who have a hereditary background of breast malignancy and the dangerous PALB2 alteration are at even greater risk, 58%.^53^ Several Asian racial groups are more likely to develop HER2‐positive breast carcinoma.^54^ Compared to more prevalent hormone‐receptor‐positive kinds of breast cancer, this biological subtype is more aggressive and has a worse prognosis.^55^ Thus, genetic screening may give sufficient insight into the propensity of certain cancers and may serve as a basis for regular monitoring or even prophylactic surgical or vaccine‐mediated interventions. However, such interventions are very rarely followed due to a lack of knowledge as well as social deterrents and taboos. Substantial variations were observed in 5‐year survival rates of different ethnic populations in breast cancer research that included 777 Hispanic patients, 1016 Black patients, and 4885 White patients. Patients of Hispanic descent had survival rates of 70% ± 2%, Black patients of 65% ± 2%, and White patients of 75% ± 1%.^56^ These variations are reflective of the stage of diagnosis as the principal factor. The percentage mortality rate for US patients for different stages of breast cancer is shown in Figure 3A and indicates that early detection irrespective of racial bias can lead to complete recovery in most cases.

According to estimates, there were 654620 individuals in the United States who have a background of pulmonary carcinoma, and another 236740 incidents have been discovered in 2022. The percentage mortality rate for US patients with respect to different stages of lung cancer is shown in Figure 3B. For therapeutic reasons, small cell lung cancer (SCLC; 14% of incidents) and non‐small cell lung cancer (NSCLC; 82% of incidents) are two different types of lung cancer, with around 3% of cases having undetermined histology. The emergence of targeted cancer medicines has irrevocably altered the therapeutic scenario for NSCLC, making personalized treatment for lung carcinoma (i.e., NGS) the treatment of choice. In subpopulations of NSCLC sufferers who are eligible for this therapy, the clinical implementation of specific kinase medicines has increased the survival rate significantly.^32^ ALK rearrangement, EGFR mutation, and PDL‐1 testing are all recommended as part of molecular testing for metastatic NSCLC by NCCN in light of the significance of directed treatment for the overall control of lung cancer. As part of a comprehensive molecular profile, screening for mutations in BRAF, KRAS, RET, NTRK1/2/3, METex14 skipping, and ROS1 should also be done. It is crucial to take into account how race may affect biomarker screening and molecular analysis in lung cancer because these techniques are increasingly vital for improving cancer outcomes for individuals with NSCLC. Although targeted treatments were previously acknowledged as improvements in the management of NSCLC, it was highlighted that the distribution of their use across racial and socioeconomic strata was uneven.^57^ One early research revealed that individuals with low incomes and those who lived in highly impoverished places were less probable to have EGFR screening. African Americans underwent lower rates of erlotinib and EGFR screening in univariate analysis than Whites, even after excluding the effects of socioeconomic, clinical, and demographic variables.^57^ Other research has looked at the correlation between the probability of ordering an EGFR test and institutional and geographical features of the treating hospital as well as differences in socioeconomic determinants. It was shown that if the region had a wealthier or more educated population, hospitals were extra inclined to seek EGFR screening for individuals having advanced NSCLC.^58^ Lynch et al. emphasized that there have been long‐standing issues with getting anti‐EGFR treatments and EGFR screening in regional hospitals, raising concerns that this might exacerbate the imbalance in cancer inequalities.^58^ Although there were no racial differences in the frequencies of EGFR alterations and ALK rearrangements, individuals from most impoverished nations remained with a lower probability of ever having had any type of biomarker examined. The frequency of thorough genetic testing with NGS was even lower in all populations around the world. Compared to white patients, black patients had a lower likelihood of having had NGS analysis (39.8% vs. 50.1%, p 0.0001).^59^

More than 1.4 million males and females were anticipated to have received a colorectal carcinoma diagnosis as of January 1, 2022 and 151030 more patients are anticipated to get the diagnosis this year. KRAS mutations are present in around 45% of colorectal malignancy (CRC) patients.^60^ About 12% of CRCs have a BRAF alteration (V600E), which is connected to a worse prognosis.^61^ The percentage mortality rate for US patients with respect to different stages of breast cancer is shown in Figure 3C and is indicative of the fact that late detection results in a very high probability of fatal consequences, thus necessitating the emphasis on biomarker screening and early detection.

One of the most inherited cancers is prostate cancer which can be easily diagnosed at early stages by marker‐based screening.^62^ Incidents of prostate cancer strike one in nine American men throughout the course of their lifetimes. However, this ratio is one in seven for Black males, whose mortality rate is 1.7 times higher than their white counterparts.^9^ The mortality rate for prostate cancer concerning different stages of cancers in different racial cross sections of patients is shown in Figure 3D. Notably, in prostate cancer, there is a significantly low mortality rate at the regional and localized stages as compared to other types of cancer. Prostate cancer is generally curable if detected early when the cancer is at a localized or regional stage. Black patients, however, often come for treatment only in the advanced stages of the disease and have high PSA values.^63^ Black men had lower PSA screening rates than White men.^64^, ^65^ A similar scenario is seen in African men where lower incidence (Figure 1) is only reflective of poor screening and unfortunately results in a high mortality rate (Figure 2) as a result of late diagnosis. Due to advances in screening technologies, more than 50% reduction in prostate cancer occurrence has been seen since 1992 with an increase of more than 2% in overall survival rates.^66^

## 4 DISPARITIES IN TREATMENT

**FIGURE 4:**
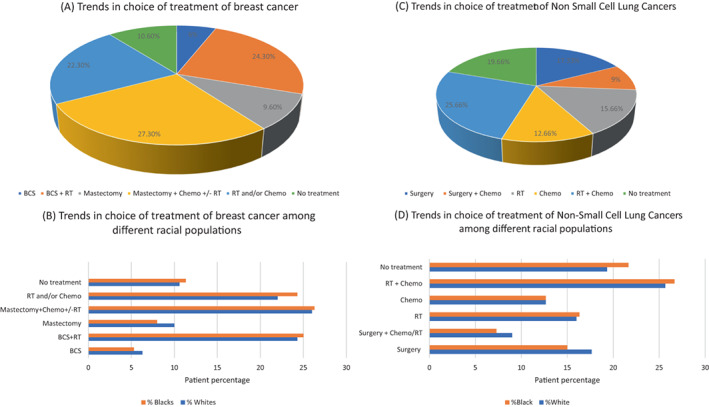
Trends in the choice of treatment for various cancers is a reflection of racial biases. (A) Trends in choice of treatment of breast cancer, (B) Different racial population show differences in their choice of treatment for breast cancer among US patients, (C) Trends in choice of treatment of non‐small cell lung cancers, and (D) Different racial population show differences in their choice of treatment for non‐small cell lung cancers among US patients. Orange shows the population percentage of Black patients for a particular type of treatment and blue shows the population percentage of White patients. *Source*: Data were obtained from a study by Miller et al.^72^

Due to population expansion as well as improvements in early identification and treatment, there are more cancer survivors than ever before. As of Jan 1, 2022, over 4000000 females in the United States were projected to have a background of metastatic breast carcinoma, and an additional 287850 females will receive a new diagnosis. According to a study, three‐fourths of the 150000 approx. breast carcinoma survivors who have the metastatic illness, are survivors who were detected early and initially confirmed at cancer stage I, II, or III.^67^ More than 2.7 million females that are two‐thirds of breast carcinoma survivors are 65 years of age or older, while just 6% are under 50. While one‐third (34%) of females with stage I and stage II carcinoma receive mastectomy, frequently without chemotherapy or radiation, the remaining half of these women choose breast‐conserving surgery (BCS) plus adjuvant radiotherapy. In contrast, 65% of females with stage III carcinoma, elect a mastectomy as their therapy of choice and in addition typically get chemotherapy. For stage I and stage II illness, Black females are less probable than White females to undergo BCS (60% vs. 64%, respectively). Black females are more probable to have just chemotherapy and/or irradiation for stage III illness (9% vs. 6%) and are less certain to undergo excision (57% vs. 66%). Sixty percent of female individuals with metastatic illness (stage IV) get just irradiation or chemotherapy. Adjuvant hormonal treatment is administered to at least 50% of females with invasive breast carcinoma who have tumors that express hormone receptors and who do not undergo carcinoma‐targeted surgery, chemotherapy, or irradiation. Some BCS‐eligible females choose to have surgery because they are reluctant to receive irradiation therapy, dread a recurrence, or have a medical condition that makes it impossible for them to receive irradiation.^68^, ^69^, ^70^ Trends in the treatment of breast cancer are shown in Figure 4A,B. The accessibility of conveyance and/or the proximity to the treatment site might be structural barriers to undergoing irradiation therapy.^71^ Black females are less inclined toward diagnosis at stage I carcinoma than White females that is 53% versus 68% of cases which leads to lower survival rates of black females for all stages, with the highest discrepancy for advanced malignancy that is 65% versus 77% for stage III and 19% versus 30% for stage IV.

Among NSCLC patients having stage I or II NSCLC, over 55% have surgery that involves wedge resection, sleeve resection, lobectomy, or pneumonectomy. Wedge resection involves the elimination of some part from a lung lobe; sleeve resection involves the elimination of the tumor plus a section of the damaged air track. In comparison, approximately one‐fifth of patients with NSCLC stage III are able to receive surgical intervention; the majority (61%) receives chemotherapy and/or radiation therapy. Blacks are substantially lesser inclined than Whites to undergo surgery—16% versus 22% for stage III and 49% versus 55% for stages I and II. In comparison to whites, the (10%), frequency of receiving therapy in blacks (15%) is low in stages I and II illness. There is mixed evidence regarding whether, Black patients who receive platinum‐based chemotherapy have a worse treatment outcome or more severe toxicity, which may influence survival, coupled with lower post‐operative death rates. Trends in the treatment of lung cancer are shown in Figure 4C,D which compares the choice of treatment in different ethnic groups. Although it has been seen that surgery remains to be the choice of treatment, it is not preferred by most African Americans.

The majority of patients surviving colorectal carcinoma, involving both genders belong to the age group of 65 and above. About 67% of individuals with colorectal carcinoma (stage III) get chemotherapy involving adjuvant to discourage the chances of recurrence, compared to the bulk of patients at stage I & II colorectal carcinoma (84%), who undergo partial surgical removal of colon, avoiding chemotherapy. Proctectomy and related procedures are the most prevalent therapy for individuals suffering from rectal cancer stage I (61%) and almost half additionally get neoadjuvant irradiation or chemotherapy. Surgical plus neoadjuvant chemotherapy and irradiation treatment are often used to treat rectal tumors in stages II and III. Individuals having stage IV colon carcinoma (49%) and rectal carcinoma (29%), respectively, typically have surgery along with radiation and/or chemotherapy. Rectal cancer treatment differences between races are far higher than for colon carcinoma, which is probably due, at least in part, to the more complicated nature of care management. For both initial‐stage colon and rectal malignancies, Black individuals are lesser inclined than White individuals to have surgery with the gap being substantially bigger for rectal carcinoma than it was previously noted for colon cancer.^73^, ^74^ Proctectomy or proctocolectomy is significantly less common for Black individuals with stage I rectal carcinoma than for White individuals (41% against 66%). While 7% of Blacks are unable to get any treatment, this figure is 3% for whites. Fifty‐seven percent of Stage II/III black patients receive neoadjuvant chemo‐radiotherapy before proctectomy or proctocolectomy as contrasted with 60% of White patients. The unavailability of skilled practitioners also contributes to treatment disparities. For example, there is fewer than one pathologist for every 500000 people in sub‐Saharan Africa.^75^ There are not enough trained cancer surgeons, less than two surgeons are available for every 100000 people. These ratios are significantly lower than the American average of 35 surgeons per 100000 people and one pathologist for every 15000 people.^76^

In the United States, there were more than 3.5 million males who have had prostate cancer previously, and in 2022, there might be 268490 new cases identified. Eighty‐five percent of male prostate cancer survivors are above 65 years of age, while only 1% (12630) are under the age of 50. Active monitoring of low‐risk illness climbed from 15% in 2010 to 42% in 2015^77^ according to the National Comprehensive Cancer Network 2010 publication. This recommends toning down the excessive treatment,^78^ whereas radical prostatectomy decreased from 47% to 31%. Numerous studies indicate a rise in proactive monitoring among elderly males aged 75 years and above, making them prone to early detection and 100% cancer regression.^79^ However, although there is not much racial discrepancy as per genetic predisposition, regular screening remains to be the only determinant between 100% cancer recovery versus fatal outcome upon late diagnosis.^80^, ^81^, ^82^ Hence the greatest solution to the racial discrepancy in its mortality lies in greater awareness and screening outreach measures that need to be implemented on a global scale.^83^

## 5 DISPARITIES IN TARGETED TREATMENTS AND IMMUNOTHERAPY

Cancer health disparities have continued to increase despite advancements in treatment strategies. Cost and availability are the major factors that widen this gap.^84^ Immunotherapy is currently regarded as a regular part of the first and foremost therapy for metastatic tumors which lack targetable mutations.^85^ Mortality due to cancer has been reduced significantly due to the elevated use of immune checkpoint blockers. Disparities in targeted treatments like immunotherapy are seen even at the stage of recruitment for their clinical trials. Despite the fact that black populations have more cases of lung carcinoma, only 4.5% of blacks participated in screening trials^86^ and 2% in the durvalumab trial for stage III NSCLC.^87^ A retrospective cohort study using NCDB 2004–2012, also found a huge difference in their study sample among blacks who have opted for immunotherapy for melanoma, 97.7% less than whites.^88^ A recent study shows the disparity in pembrolizumab trials for breast cancer patients, where approximately 12 white females have participated as opposed to only 1 black female patient for the immunotherapy trial.^89^ In metastatic HCC, immunotherapy is preferred over chemotherapy for survival in general. Early accessibility to immunotherapy is characterized by major differences among Hispanics and Blacks as compared to Whites.^90^

PDL1 expression and tumor genomic profiles in breast, lung, and colorectal cancers have not been shown to differ in different ethnic groups, although there are notable variations in the immune cell population in the tumor microenvironment.^91^ For example, compared to non‐Hispanic white patients, breast tumors from black patients had a compelling immune cell prevalence and enhanced expression of inhibitory receptors like PD1, CTLA4, and LAG3.^92^ Similar trends were also seen in prostate tumors for increased expression of proinflammatory genes among this population.^93^ All these findings suggest better immunotherapeutic options for black patients but the reality is contrasting, due to patient‐level factors (SES, behavior toward treatment and ethnicity, etc.), provider‐level factors (cost of immunotherapy, knowledge, beliefs, and attitude toward patient, etc.) and system level factors (reimbursement and infrastructure quality, etc.).

Regulatory bodies play a role in specialized therapies like immunotherapy. It has been found that the percentage of patients having immunotherapy before and after approval by the FDA is increased to 12.4% in NSCLC.^94^ Immune checkpoint inhibitor use has increased exponentially over the past 10 years, significantly decreasing carcinogenicity‐based deaths. However, overall cancer registration among populations indicates the advantage of targeted treatments in non‐Hispanic Whites, as compared to other minority subgroups. Contrary to popular belief, Asian lung cancer patients seemed to survive better as compared to other ethnic populations.^95^

## 6 DISPARITIES IN CLINICAL TRIAL INVOLVEMENT

Clinical trials with sound design have improved the diagnosis and treatment of cancer. Cancer treatment is showing new innovations every day due to extensive improvements in our scientific knowledge. However, the implementation of novel treatment strategies requires the informed consent of patients, participating in clinical trials. Statistics reveal that less than 5% of cancer patients are confident enough to do so.^96^, ^97^, ^98^, ^99^ A meta‐analysis revealed a somewhat better level of participation of 8% of patients in the case of industry‐sponsored projects,^100^ possibly due to perks given by industries but at the same time the recruitment of patients for trials by the industry is largely from academic centers whereas the proportion of patients in investigator‐initiated trials is from community centers.^84^ Geographical accessibility to a clinical study may affect its enrollment. There is evidence that unequal geographical accessibility to health care is correlated to adverse consequences and inferior quality of life as well as inadequate treatment compliance.^101^ Syed et al.^102^ have further demonstrated that minorities and individuals with lower incomes are disproportionately impacted by these differences. This hinders equitable representation in therapeutic studies as well. Only 37% of people with cancer in Pennsylvania who participated in a nationwide poll said they would commute to take part in a clinical trial.^103^ Similar results were shown by Lara et al. in a prospective analysis of cancer patients being conducted at the University of California Davis Cancer Center, where the second most frequent explanation given for not participating in a trial was the patient's remoteness from the study center.^104^ Recent research showed that even in the United States, clinical trials are not equally accessible.^105^ According to a study by Galsky et al., 38.4%, 45.6%, 50.2%, and 52.2%, respectively, of patients with NSCLC, breast, prostate, and colorectal cancers need to travel more than an hour to reach a trial location.^106^

Phase 3 prostate cancer studies in the United States had a significant underrepresentation of Black males between 1987 and 2016; of the 72 clinical trials examined, 83.4% of the males who enrolled were White, compared to 6.7% who were Black.^107^ In lower and middle‐income nations throughout the world, the difference in access to trials for cancers is more pronounced. Only 1951 trials were available in lower and middle‐income nations, compared to approximately 4700 trials in high‐income nations for lung, breast, and cervical malignancies.^108^ For instance, there are not many cancer clinical studies available in India, despite the significant patient burden. Only 350 interventional studies were filed between 2007 and 2017 according to a recent CTRI‐listed audit of trials.^109^ According to Carneiro et al., there are between 0.14 and 10.7 interventional trials per 100000 people in Europe.^110^ African Americans make up about 5% of cancer clinical study enrollment. The execution of and accessibility to preventative clinical trials are likely to be impacted by the socioeconomic disparity and multiculturalism of the country.^111^ Major issues responsible for this disparity included inadequate patient rights protection and compensation for the harm resulting from clinical trials, poor compliance with informed consent protocol, inadequate scientific and ethical review processes, subpar regulatory procedures for new drugs, and, most importantly, lack of post‐trial cohort population's access to prohibitively expensive cancer treatments that had been demonstrated to be effective in low‐ and middle‐income country settings. According to Agarwal et al.^112^ and Joseph et al.,^113^ there are a number of obstacles in conducting these researches, including the workforce's mobility, socioeconomic difficulties (such as gender inequality, casteism, and sickness stigma), and an absence of availability of primary healthcare facilities in low‐ and middle‐income countries.

For NSCLC‐focused treatment studies, genomic analysis is frequently a requirement for inclusion; as a result, disparities in comprehensive molecular profiling/NGS analysis may be significant in discrepancies in trial enrollment among racial groups. Recent research has focused on whether racial inequities exist in the use of biomarker screening and if inclusion in clinical trials is correlated with thorough genetic testing. Importantly, individuals were considerably more likely to take part in a clinical study if their tumors had undergone NGS analysis,^59^ since such marker profiling gave greater promise of favorable outcomes, as evidenced due to targeted therapies.

## 7 DISPARITIES IN PALLIATIVE CARE

Palliative care simply means “active overall care for patients whose conditions don't improve with treatment.”^114^ The effectiveness of pain treatment and the use of hospice care are major tenets of discrepancies based on socioeconomic factors and the availability of medical facilities. Nearly 58% of the world has palliative care facilities,^115^ but they are not similar in all regions. USA, Australia, Europe, and Canada have modern facilities whereas South American and African regions are devoid of similar services.^115^ While there are some similarities in palliative cancer care worldwide, significant differences exist in the prevalence, knowledge, and accessibility of palliative care facilities. There is often a lack of integrated cancer‐specific treatments into care and cultural considerations that necessitate a customized approach to treatment.^116^ A study found that obtaining any palliative care was considerably less likely at hospitals that served impoverished communities.^117^ Non‐Hispanic Blacks continue to be under‐represented among hospice patients despite improvements in the accessibility of hospice care.^118^ Despite a 14% increase in a hospice facility, an analysis of 204175 hospitalizations with late‐stage cancer found that non‐Hispanic Blacks were significantly less probable than their White counterparts to avail hospice care in terminally ill patients.^119^ When palliative and hospice care among 133 non‐Hispanic Black and White patients at a cancer treatment center were compared, non‐Hispanic Blacks were found to have considerably lower levels of state‐of‐the‐art facilities than Whites. A comprehensive study evaluating the effectiveness of cancer pain therapy studies conducted in North America, Europe, and Africa was published by Odonkor and colleagues.^120^ Only 3 of the 18 studies were conducted in Africa (Egypt), and the investigators highlighted the uneven distribution of trials worldwide.^120^ Only 41.4% of the respondents in a survey of 15 Middle Eastern nations said their organization had a palliative care facility.^121^ The first step toward reducing inequities in the usage of appropriate treatment for cancer patients is increased knowledge and uniform accessibility of hospice & palliative care.^122^ Reluctance to utilize hospice care more frequently is mostly due to: (1) Prohibitive costs; (2) Cultural or personal values at variance with modern hospice concept; (3) Ignorance of hospice care; (4) Absence of trust in the medical care; and (5) Reluctance of engaging financial burden of palliative care, especially in terminal conditions.^123^ Both, gaps in critical disease treatment and inequities related to palliative care must be understood to eliminate the disparity. Analysis of 187 individuals who underwent hospitalized palliative care at a hospital revealed that location of birth and racial group were strongly linked with disposition.^124^

Studies have also revealed that minority groups, such as African Americans, Asian Americans, and Hispanics/Latinos seek hospice care less frequently.^125^ Cultural variations in the impact of the disease or its mitigation and palliative care among different sections of patients and their families are also often variable in the western world as compared to close‐knit societal frameworks as seen in South Asia, Southeast Asia, and the far East. Buddhism's predominant faith in “natural fate” urges sufferers to face pain as they await death. Since Buddhism predominates in China and Southeast Asia, there are reluctances to palliative hospice care.^126^ In many countries, talking about cancer or palliative care is fraught with cultural taboos and fears of the disease.^127^ Some ethnic groups continue to believe that cancer is infectious, especially in some regions of Africa^128^ which has made it difficult to manage palliative treatment and has led to the isolation of patients due to social stigma.^129^ This societal outlook toward palliative care also must be acknowledged and weighed against scientific rationale with compassionate patient management and the spread of awareness. State‐of‐the‐art palliative treatment techniques need to be more uniformly distributed to ease pain and suffering, notwithstanding racial discrimination globally.^130^ To evaluate and eradicate racial/ethnic inequities in hospice and palliative care, investigational strategies are required and the financial burden needs to be effectively managed.

## 8 CONCLUSION

Growing industrialization is often associated with drastic changes in diet, lifestyle, and socio‐economic conditions of people resulting in skewed incidences of certain types of cancers in a disparate manner around the world. Cancer care and novel treatment strategies have indeed resulted in a significant reduction in mortality rates of certain cancers and have also made preventive interventions possible. Despite significant advancements in our knowledge of certain biomarkers and their regular screening having an immense impact on cancer outcomes and mortality rates, inequalities exist in global populations sheerly due to late diagnosis. The prohibitive cost of treatment and unequal distribution of state‐of‐the‐art hospice facilities and trained medical staff are significant causes of disparities in cancer treatment and global mortality rates. More studies are recommended to further streamline meticulous data collection of the medical system, genetic, and sociocultural environment in order to better identify and comprehend the pertinent levels of arbitration needed to reduce and finally eradicate cancer‐related health discrepancies. However, just an increased understanding of its reasons, may not suffice alone to eradicate cancer health inequalities. To better understand the etiology of cancer and develop effective therapies oriented toward specific ethnicities, effort needs to be undertaken for acquiring genome analysis data sets and combinatorial therapeutics available in a broader scope of ethnic populations. Biomarker evaluation and prophylactic measures for high‐risk groups compounded with regular screening are crucial for the ability to detect cancer at an early stage. As has been clearly shown by our study, despite the availability of state‐of‐the‐art therapeutic options, late detection of cancer can in most cases adversely impact the cancer outcome. Thus early detection of most cancers can significantly lower mortality rates, irrespective of ethnicity necessitating more aggressive regular screening initiatives all over the world. In the case of certain cancers like prostate cancer, it may even result in 100% regression across all ethnic populations, provided the early diagnosis is effectuated. Prophylactic vaccination in certain cancers is not widely accepted due to social taboos which can be mitigated only by better education and awareness. It is crucial to broaden ongoing cultural and linguistic programs directed toward cancer awareness and broaden our outreach for better cancer management. The institutional elements and regulations that enable behavioral changes, such as tobacco control, should also be supported. Most crucially, government‐driven schemes for improvements that support health equity, ubiquitous insurance policies, and availability of standard treatment for all must be ensured if the aforementioned disparities are to be erased.

## AUTHOR CONTRIBUTIONS

**Bharmjeet:** Data curation (supporting); investigation (supporting); writing – original draft (supporting). **Asmita Das:** Conceptualization (lead); supervision (lead).

## CONFLICT OF INTEREST

The authors have stated explicitly that there are no conflicts of interest in connection with this article.

## ETHICS STATEMENT

The present work has been done in compliance with the ethical guidelines of Delhi Technological University.

## Data Availability

Data sharing is not applicable to this article as no new data were created or analyzed in this study.
